# Targeting of LRRC59 to the Endoplasmic Reticulum and the Inner Nuclear Membrane

**DOI:** 10.3390/ijms20020334

**Published:** 2019-01-15

**Authors:** Marina Blenski, Ralph H. Kehlenbach

**Affiliations:** Department of Molecular Biology, Faculty of Medicine, Göttingen Center of Biosciences (GZMB), Georg-August-University Göttingen, Humboldtallee 23, 37073 Göttingen, Germany; Marina.Blenski@med.uni-goettingen.de

**Keywords:** inner nuclear membrane, tail-anchored proteins, LRRC59, TRC40

## Abstract

LRRC59 (leucine-rich repeat-containing protein 59) is a tail-anchored protein with a single transmembrane domain close to its C-terminal end that localizes to the endoplasmic reticulum (ER) and the nuclear envelope. Here, we investigate the mechanisms of membrane integration of LRRC59 and its targeting to the inner nuclear membrane (INM). Using purified microsomes, we show that LRRC59 can be post-translationally inserted into ER-derived membranes. The TRC-pathway, a major route for post-translational membrane insertion, is not required for LRRC59. Like emerin, another tail-anchored protein, LRRC59 reaches the INM, as demonstrated by rapamycin-dependent dimerization assays. Using different approaches to inhibit importin α/β-dependent nuclear import of soluble proteins, we show that the classic nuclear transport machinery does not play a major role in INM-targeting of LRRC59. Instead, the size of the cytoplasmic domain of LRRC59 is an important feature, suggesting that targeting is governed by passive diffusion.

## 1. Introduction

The nuclear envelope comprises three continuous membrane regions: the outer nuclear membrane (ONM), which is very similar in protein composition to the membrane of the endoplasmic reticulum (ER), the inner nuclear membrane (INM), which contains a distinct set of proteins as compared to the ONM, and small membrane patches at the level of the nuclear pores, where ONM and INM are connected. Several hundred INM-proteins have been identified, mostly by proteomic screens [1,2]. Two major models try to explain the selective targeting of such proteins to the INM (reviewed in references [3,4,5,6]). The “active transport model” is based on similar principles to those established for the transport of soluble proteins into or out of the nucleus. Here, cargo proteins contain nuclear localization signals (NLSs) or nuclear export sequences (NESs) that bind to soluble transport receptors belonging to the family of importin β-related proteins, also referred to as karyopherins. These proteins transiently interact with certain nucleoporins, the proteins of the nuclear pore complexes (NPCs), mediating translocation through the nuclear pores. The best-described nuclear import pathway is the importin α/β-dependent pathway, where importin α serves as an adaptor protein that interacts with proteins carrying a classic NLS (cNLS) and also with importin β. Like other import receptors, importin β can also bind directly to a cargo molecule, independent of importin α [7,8,9]. Dissociation of soluble transport complexes on the nuclear side of the NPC is controlled by the small GTP-binding protein Ran, which, in its GTP-bound form, interacts with all karyopherins [10].

At the level of the NPC, soluble transport complexes travel through the central channel, where nucleoporins with characteristic phenylalanine-glycine (FG-) repeats form a permeability barrier that can be overcome by karyopherins [10,11]. Several membrane proteins of the INM were initially found to contain sequences that resemble the typical cNLS [12]. When expressed as soluble proteins lacking a transmembrane domain (TMD), fragments of the lamin B receptor (LBR) and of emerin, two well-characterized proteins of the INM, accumulated in the nucleus [13,14,15], suggesting the functionality of the presumed localization signals. Based on such observations, a typical INM-protein was thought to integrate into the membrane system of the ER, expose an NLS to its cytoplasmic side for binding to a nuclear transport receptor, and travel along the ONM and the membrane patch within the NPC to the INM. This model received some support from results obtained in the yeast *S. cerevisiae*, where INM-localization of the membrane proteins Heh1 and Heh2 depends on NLSs, the karyopherins Kap60 and Kap95 (i.e., the yeast homologues of mammalian importin α and importin β), as well as the Ran system [16]. In addition, an unfolded linker between the TMD and the NLS is required for efficient localization of the reporter proteins to the INM [17]. A similar mode of transport has been suggested for the mammalian nucleoporin Pom121 [18]. For emerin and LBR, however, nuclear transport receptors seem to be dispensable [19,20], despite the presence of NLS-like sequences.

**Figure 1:**
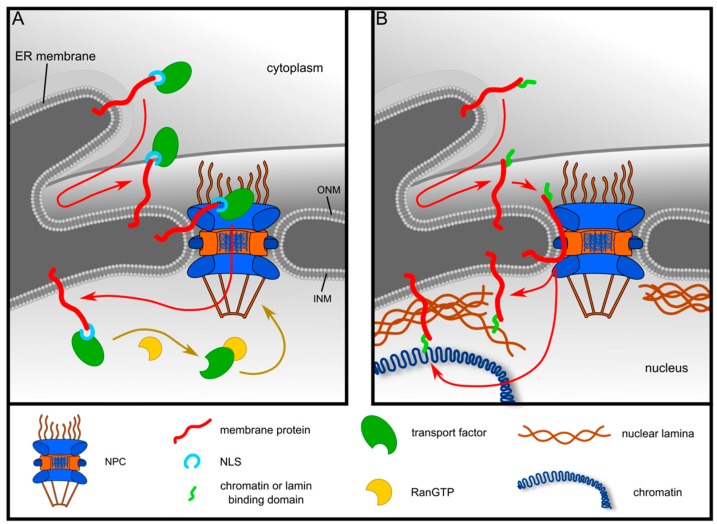
Models for targeting of membrane proteins to the inner nuclear membrane. (**A**) Active-transport-model. After insertion into the endoplasmic reticulum (ER), proteins expose a nuclear localization signal (NLS) to the cytoplasm, which can be recognized by nuclear transport receptors. After translocation from the outer nuclear membrane (ONM) to the inner nuclear membrane (INM), the complex is dissociated by nuclear RanGTP. (**B**) Diffusion-and-retention model. Membrane proteins diffuse passively through the nuclear pore complex (NPC) and are sequestered at the INM upon interaction with the nuclear lamina and/or chromatin. See text for details. Modified after reference [29].

In contrast to the “active transport model”, the “diffusion-and-retention model” [21] suggests that transport of proteins to the INM *per se* does not involve active, signal-, receptor- or energy-dependent steps. Instead, membrane proteins are thought to passively diffuse from the ER via the ONM to the INM. There, they interact with nuclear lamins, intermediate filament proteins that underlie the INM, and/or with lamina- or chromatin-associated proteins. Thus, retention at a defined position results in accumulation of INM-proteins at their final destination. Instead of the central channel, peripheral channels could mediate passage of membrane proteins to the nuclear side of the NPC [22,23]. An energy-requirement for INM-targeting could result from nucleotide-dependent effects on protein mobility within the ER-membrane [20] and/or from ATP-dependent restructuring of the NPC [24]. Several observations support the diffusion-and-retention model: First, INM-targeting of emerin depends on the presence of lamin A/C within the nuclear lamina [25]. Second, a size limit has been observed for nuclear domains of INM-proteins [20,24,26,27]. Furthermore, a strong bias towards small nucleoplasmic (i.e., initially cytoplasmic) regions compared to luminal regions was found in a dataset of a large number of INM-proteins, but not of other membrane proteins [6]. Third, an in vitro system for the analysis of protein targeting to the INM revealed that classic inhibitors of nucleocytoplasmic transport do not affect trafficking of several reporter proteins to the INM [20]. Fourth, knockdown of 17 members of the importin α/β-families did not result in a significant reduction of INM-targeting of an LBR-based reporter protein [19]. The diffusion-and-retention model was further supported by mathematical modeling, assuming mobile and immobile pools of proteins in the ER, the ONM and the INM, respectively [20]. It may be valid for the majority of INM-proteins, whereas the active-transport model may apply for a subset of proteins, particularly in yeast [5,6,28]. The two models are depicted in Figure 1.

**Figure 2:**
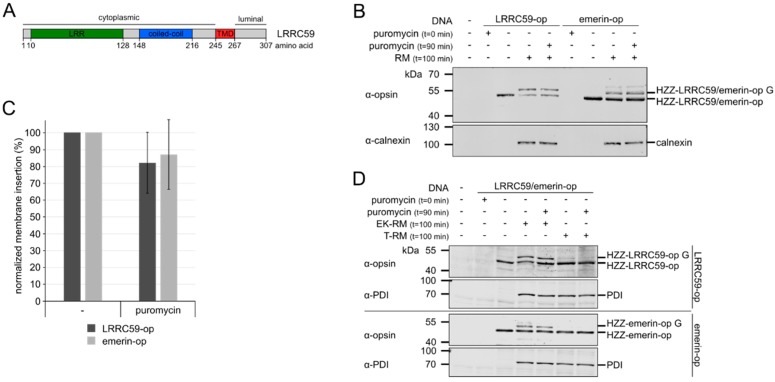
Posttranslational membrane insertion of LRRC59. (**A**) Schematic representation of full length LRRC59. The leucine rich repeat region (LRR), the putative coiled-coil domain and the transmembrane domain (TMD) are indicated. (**B**) Opsin-tagged LRRC59 (HZZ-LRRC59-op) and emerin (HZZ-emerin-op) were produced in vitro by coupled transcription-translation reactions in the absence (−) or presence (+) of rough microsomes (RM) and with (+) or without (−) the addition of puromycin. Proteins were analyzed by SDS-PAGE, followed by immunoblotting using antibodies against the opsin-tag and calnexin as loading control. G indicates the glycosylated forms of LRRC59-opsin and emerin-opsin. (**C**) Quantification of the results in (**B**). The error bars indicate the standard deviation from the mean of six independent experiments. (**D**) Membrane insertion of HZZ-LRRC59-op or HZZ-emerin-op after in vitro transcription-translation reactions into EDTA/high-salt (EK-RM) or trypsin-treated (T-RM) microsomes with (+) or without (−) addition of puromycin. Proteins were analyzed by SDS-PAGE, followed by immunoblotting using antibodies against the opsin-tag and PDI as loading control.

LRRC59 (leucine-rich repeat-containing protein 59; p34) was originally identified as a ribosome-binding protein that also interacts with fibroblast growth factors [30]. It was reported that LRRC59 promotes importin α/β-dependent nuclear import of fibroblast growth factor 1 [31] and also of the cancerous inhibitor of PP2A (CIP2A, [32]). LRRC59 is a membrane protein containing a 244-amino acid cytoplasmic/nucleoplasmic region, a TMD close to the C-terminus, and an ensuing 40-amino-acid-region that should reside in the ER-lumen (Figure 2A). Proteins with a single TMD at the C-terminus (tail-anchored (TA-) proteins) typically require post-translational mechanisms for membrane insertion [33,34]. For LRRC59, the sequence after the TMD could be just long enough to allow co-translational insertion into the ER-membrane via the classic signal recognition particle- (SRP-) dependent pathway [35]. Alternatively, LRRC59 might use a post-translational mechanism for membrane insertion, e.g., the TRC-pathway [36,37], which is also used by typical TA-proteins like Sec61β [38,39] or emerin [40]. In this pathway, TRC40 (transmembrane domain recognition complex protein of 40 kDa, also known as ASNA1; guided entry of tail-anchored proteins 3 (Get3) in yeast), binds to the hydrophobic stretch of amino acids at the C-terminal end of TA-proteins, keeping them soluble [38,39]. Two receptor proteins at the ER-membrane, WRB (tryptophan-rich basic protein; Get1 in yeast) [41] and the mammalian-specific protein CAML (Ca^2+^-modulating cyclophilin ligand, also known as CAMLG [42]) then initiate integration of the TA-proteins into the lipid bilayer [43].

By immunofluorescence, LRRC59 was found at the ER and also at the nuclear envelope, a localization that was suggested to depend on the nuclear import receptor importin β [31]. Targeting of LRRC59 to the INM, however, has not been demonstrated so far. 

We now provide evidence that LRRC59 can use post-translational mechanisms for membrane insertion, using a pathway that does not require the TRC40-system. Using a rapamycin-based reporter assay, we show that LRRC59 is targeted to the INM. In contrast to previous observations [31], INM-localization does not depend on the importin α/β-system. Instead, the size of the cytoplasmic domain seems to control the diffusion of LRRC59 to the INM. 

## 2. Results

### 2.1. LRRC59 can be Post-Translationally Inserted into Microsomal Membranes

Typical TA-proteins contain a single TMD very close to their C-terminus and use post-translational mechanisms for ER-insertion [34]. LRRC59 lacks a signal peptide and contains a 40 amino acid long region behind its TMD (Figure 2A). To test if LRRC59 can be post-translationally integrated into ER-membranes, we used a microsome insertion assay [41,44,45], where the protein of interest is produced in an in vitro transcription/translation system. Insertion into microsomal membranes is monitored by the detection of N-linked glycosylation of a 13-amino acid opsin-tag at the very C-terminus of the protein, a modification that is specific for the microsomal lumen. As a control, we used emerin, an established TA-protein of the INM [40]. As shown in Figure 2B, LRRC59-opsin and emerin-opsin were produced in reticulocyte lysates. The addition of microsomes after the in vitro transcription/translation reaction resulted in slower migrating form of the proteins, indicating glycosylation of the opsin-tag and, thus, insertion into the microsomal membranes. To demonstrate that the observed shift in molecular weight resulted from glycosylation, we added the enzyme peptide-N-glycosidase F (PNGase F) after the reaction. Indeed, a single band was observed under these conditions, indicating de-glycosylation of LRRC59-opsin by PNGase F (Figure S1A). As a test for post-translational membrane insertion, the translation inhibitor puromycin was added 90 min after the start of an in vitro transcription/translation reaction. After 10 additional minutes, rough microsomes (RM) were added to allow post-translational membrane insertion of the reaction products. For both proteins, puromycin resulted only in a small reduction of the intensity of the glycosylated form, showing that membrane insertion of emerin and LRRC59 can indeed occur after translation (Figure 2B,C). Addition of puromycin at the beginning of the reaction completely inhibited protein synthesis, confirming the efficacy of the drug. 

Post-translational insertion of proteins into membranes can either occur spontaneously, as has been shown for cytochrome b5 [38,39] or in a receptor-dependent manner [46]. To distinguish between these possibilities, we treated RM with trypsin in order to remove potential receptor proteins from the microsomal membrane surface. This treatment resulted in a loss of immunoreactivity of the TRC40-receptor protein CAML, indicating digestion of its cytoplasmic region, whereas the ER-luminal protein PDI was not affected (Figure S1B). As a control, we used microsomes that had been treated with EDTA and high salt in order to remove loosely attached proteins from the membranes (EK-RM). Trypsin-treatment completely abolished the glycosylation of both emerin and LRRC59 (Figure 2D). For cytochrome b5, by contrast, two protein products were observed (i.e., glycosylated and un-glycosylated) with trypsin-treated and un-treated microsomes (Figure S1C). These results suggest that post-translational membrane insertion of LRRC59 requires auxiliary factors (e.g., a receptor protein on the microsomal surface), like that of emerin.

### 2.2. Membrane Insertion of LRRC59 Does Not Require the TRC40 System

**Figure 3:**
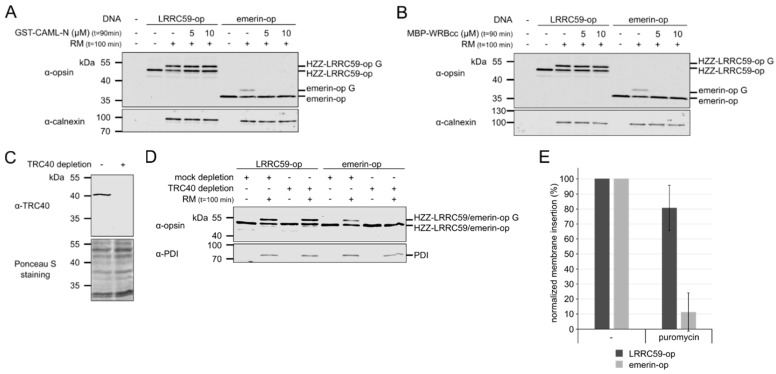
The TRC-pathway is not required for membrane insertion of LRRC59. (**A**) In vitro transcribed/translated HZZ-LRRC59-opsin or emerin-opsin was subjected to membrane integration assays using rough microsomes (RM) in the absence (−) or presence (+) of 5 or 10 µM GST-CAML-N, as indicated. Reactions were analyzed by SDS-PAGE followed by immunoblotting, using antibodies against the opsin-tag and against calnexin as loading control. Note that the emerin-construct used in this experiment lacks the N-terminal HZZ-tag. G indicates the glycosylated forms of LRRC59-opsin and emerin-opsin. (**B**) As (**A**), using MBP-WRBcc instead of GST-CAML-N. (**C**) The rabbit reticulocyte lysate used for coupled transcription-translation reactions was immunodepleted using antibodies against TRC40 (+) or IgG (−) as control. Depletion was analyzed by SDS-PAGE, followed by Western-blotting and Ponceau S- (bottom) and immunostaining using an antibody against TRC40. (**D**) Membrane insertion of HZZ-LRRC59-opsin and HZZ-emerin-opsin using TRC40- or mock-depleted lysates (**C**) for coupled in vitro transcription and translation with (+) or without (−) the addition of rough microsomes (RM), as indicated. Proteins were analyzed by SDS-PAGE, followed by immunoblotting using antibodies against PDI and the opsin-tag. (**E**) Quantification of membrane insertion reactions as in (**D**). Individual values were normalized to a reaction with mock-depleted lysate. Error bars indicate the standard deviation from the mean of five independent experiments.

TRC40 in complex with a tail-anchored protein can bind to a heteromultimeric receptor at the ER-membrane consisting of CAML and WRB [41,42,47]. For emerin, we recently showed that dominant negative fragments of either CAML or WRB inhibit post-translational membrane insertion [40]. We confirmed these results for emerin (Figure 3A,B). Membrane insertion of LRRC59, by contrast, was not inhibited by WRB- or CAML-fragments. Next, we used immunodepletion to reduce the concentration of TRC40 in the reticulocyte lysate that is used for the in vitro reactions. The efficiency of the depletion was controlled by Western-blotting (Figure 3C). Whereas TRC40-depletion strongly affected the efficiency of membrane insertion of emerin with only 11.5% remaining compared to the control lysate, that of LRRC59 was only slightly inhibited to 80.9% (Figure 3D,E). Together, these results reveal clear differences between the mechanisms of membrane insertion of emerin and LRRC59. Both proteins can be inserted post-translationally, but only emerin strictly requires the TRC-pathway. 

### 2.3. LRRC59 Localizes to the INM

**Figure 4:**
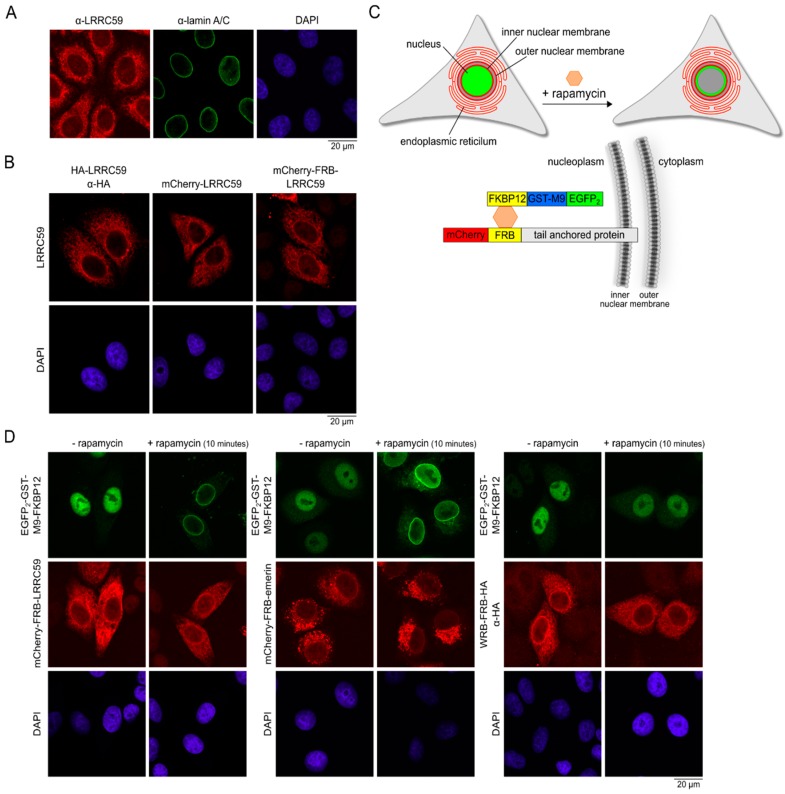
LRRC59 is targeted to the INM. (**A**) HeLa cells were grown on coverslips and analyzed by indirect immunofluorescence using antibodies against LRRC59 and lamin A/C. (**B**) HeLa cells were transfected with plasmids coding for HA-LRRC59, mCherry-LRRC59 or mCherry-FRB-LRRC59. Cells were fixed and analyzed directly (mCherry-LRRC59 and mCherry-FRB-LRRC59) or after immunostaining using an anti-HA antibody. (**C**) Schematic overview for the rapamycin-induced dimerization assay. Cells are transiently transfected with plasmids coding for the nuclear reporter EGFP_2_-GST-M9-FKBP12 (green) and the protein of interest tagged with mCherry and FRB (red). The addition of rapamycin (orange hexagon) leads to the dimerization of the FRB- and FKBP12-cassettes and to a recruitment of the reporter to the INM. (**D**) HeLa cells were co-transfected with plasmids coding for EGFP_2_-GST-M9-FKBP12 and mCherry-FRB-LRRC59, mCherry-FRB-emerin or WRB-FRB-HA, respectively, and treated with (+) or without (−) rapamycin for 10 min. Cells were fixed and analyzed directly (mCherry-FRB-LRRC59, mCherry-FRB-emerin) or after immunostaining using an anti-HA antibody (WRB-FRB-HA).

LRRC59 has previously been shown to localize to the ER as well as to the nuclear envelope [31]. High resolution microscopy, however, to distinguish between the INM and ONM has not been performed so far. First, we used confocal microscopy to confirm the localization of endogenous LRRC59 to the nuclear envelope. siRNA-depletion experiments were performed to ensure the specificity of the anti-LRRC59 antibody (Figure S2A,B). In comparison to lamin A/C, which showed a prominent localization to the nuclear rim, LRRC59 exhibited a more heterogeneous pattern with fluorescent signals at the level of the nuclear envelope as well as the ER (Figure 4A). Similar results were obtained when we analyzed the localization of various tagged forms of LRRC59 (Figure 4B).

We then used our established rapamycin-dependent dimerization assay (Figure 4C) to monitor possible targeting of LRRC59 to the INM. The assay is based on a nuclear reporter protein (EGFP_2_-GST-M9-FKBP12) that interacts with a protein of interest carrying an FRB-(FKBP-rapamycin binding) domain in a rapamycin-dependent manner [40,48]. The M9-sequence functions as an NLS that mediates nuclear import in a transportin-dependent (i.e., importin α/β-independent) manner [49]. If the protein of interest localizes to the INM, the GFP-reporter protein will be recruited to the nuclear periphery upon addition of rapamycin to the cells, resulting in a characteristic fluorescence at the nuclear rim. With this approach, we observed rapid targeting of the GFP-reporter protein to the nuclear periphery in cells expressing either mCherry-FRB-emerin or mCherry-FRB-LRRC59 (Figure 4D). In cells expressing a control protein, which should be present at the level of the ER and the ONM, but not the INM (WRB-FRB-HA), the GFP-reporter was not recruited to the nuclear periphery upon addition of rapamycin, confirming the specificity of our effects. Our results clearly show that LRRC59, like emerin, reaches the INM. Targeting is possible with the artificial sequences at the N-terminal end of the protein (mCherry-FRB), increasing the size of the cytoplasmic/nuclear region from 27.8 kDa for wild-type LRRC59 to 65.8 kDa for mCherry-FRB-LRRC59. 

### 2.4. Importin β Is Not Required for Localization of LRRC59 at the INM

The localization of emerin at the INM depends on one of its binding partners, lamin A/C [25]. We therefore depleted endogenous lamin A/C using specific siRNAs and analyzed the effects on emerin and LRRC59. As expected, in cells with reduced levels of lamin A/C at the nuclear envelope, the signal intensity for emerin at this localization was reduced and a larger portion of the protein was detected at the ER. For LRRC59, by contrast, no obvious difference between control cells and lamin A/C-depleted cells was observed (data not shown), suggesting that distinct mechanisms lead to the localization of the two proteins at the INM. 

**Figure 5:**
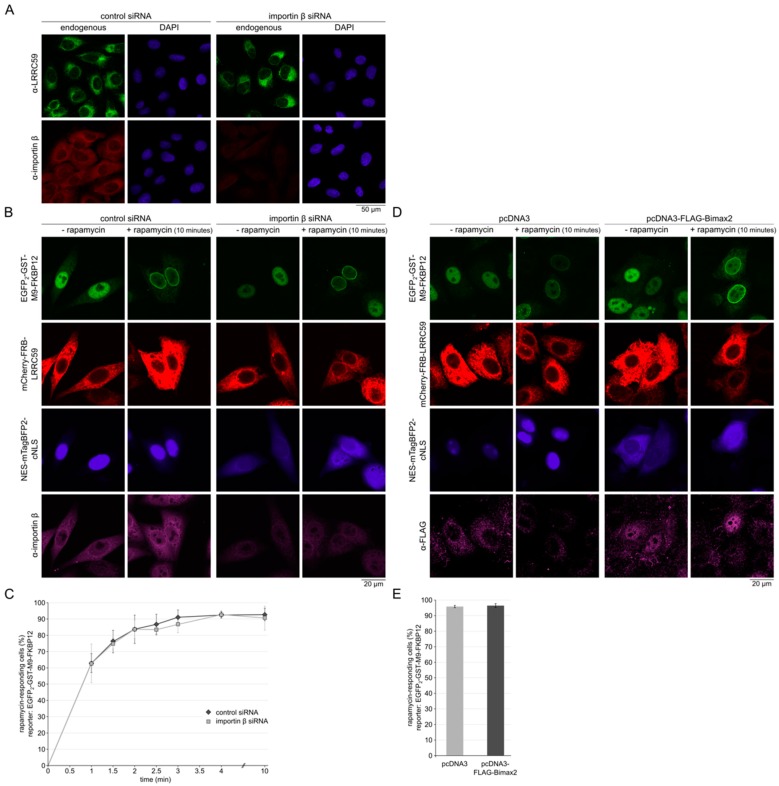
Importin β is not required for INM-targeting of LRRC59. (**A**) U2OS cells were treated with control siRNAs or siRNAs against importin β. After 48 h, cells were subjected to indirect immunofluorescence using antibodies against LRRC59 or, on separate slides, importin β. (**B**) HeLa cells were transfected with control siRNAs or siRNAs against importin β and with plasmids coding for EGFP_2_-GST-M9-FKBP12, mCherry-FRB-LRRC59 and NES-mTagBFP2-cNLS. After 48 h, cells were treated with rapamycin at room temperature for 10 min, fixed, immunostained using antibodies against importin β and analyzed by confocal microscopy. (**C**) Quantification of rapamycin assays as in (**B**). The percentage of cells responding to rapamycin with a recruitment of EGFP_2_-GST-M9-FKBP12 to the nuclear periphery was plotted against the time after addition of the drug. Error bars indicate the standard deviation from the mean of five independent experiments, counting 100 (1–4 min) or 250 (10 min) cells per time point. (**D**) HeLa cells were co-transfected with plasmids coding for EGFP_2_-GST-M9-FKBP12, mCherry-FRB-LRRC59, NES-mTagBFP2-cNLS and FLAG-Bimax2 or pcDNA3 as a control, respectively. After 48 h, cells were treated with rapamycin for 10 min, fixed, immunostained using antibodies against the FLAG-tag and analyzed by confocal microscopy. (**E**) Quantification of rapamycin assays as in (**D**), plotting the percentage of cells responding to rapamycin with a recruitment of the reporter protein to the nuclear periphery. Error bars indicate the standard deviation from the mean of 3 independent experiments, counting 200 cells each.

In a previous study, depletion of importin β with specific siRNAs resulted in strongly reduced levels of endogenous LRRC59 at the nuclear envelope [31]. First, we repeated those experiments and depleted importin β in the same cell line that had been used before, in U2OS cells. The efficiency of the depletion was monitored by Western-blotting (Figure S3A) and by immunofluorescence (Figure 5A). In contrast to previous observations, the subcellular localization of LRRC59, was not affected in importin β-depleted cells (Figure 5A), even in experiments where very high levels of depletion (e.g., with only 14% of importin β remaining) were obtained. We then extended our analysis using our established rapamycin-assay in HeLa cells that were now treated with siRNAs against importin β. Control cells and importin β-depleted cells were assayed for INM-recruitment of the GFP-reporter protein upon addition of rapamycin. To functionally control for efficient importin β-depletion, cells were co-transfected with a plasmid coding for a soluble, importin α/β-dependent transport cargo (NES-mTagBFP2-cNLS). Cells that had been treated with siRNAs against importin β showed a clear shift of this transport cargo from the nucleus to the cytoplasm (Figure 5B), indicating that importin β was rate-limiting in these cells. Rapamycin-dependent recruitment of the green reporter protein, however, was not affected in importin β-depleted cells expressing mCherry-FRB-LRRC59 (Figure 5B,C). Again, this was also observed in experiments with the highest possible level of importin β-depletion (i.e., 14.4% remaining importin β; compare Figure S3B). As an alternative method to inhibit the cellular importin α/β-pathway, we used Bimax2, an inhibitory sequence that binds tightly to importin α [50]. Cells were co-transfected with four plasmids coding for FLAG-Bimax2, NES-mTagBFP2-cNLS, EGFP_2_-GST-M9-FKBP12 and mCherry-FRB-LRRC59. As expected, Bimax2 resulted in an inhibition of nuclear import of NES-mTagBFP2-cNLS (Figure 5D). Again, rapamycin-dependent recruitment of the green reporter protein was not affected in these cells (Figure 5D,E). Together, our results show that under conditions that are still compatible with cell survival (i.e., reduction of importin β-levels to 22.8 ± 6.2% (Figure S3B) or inhibition of the import receptor by a competing protein), targeting of LRRC59 to the INM is not significantly disturbed. 

### 2.5. Nuclear Accumulation of LRRC59 Lacking the TMD

To analyze a possible active nuclear import of LRRC59 lacking the C-terminal TMD, we first expressed a GFP-tagged version (EGFP-LRRC59 aa1-244). As shown before [31], such a protein accumulated in the nucleus of transfected cells (Figure S4A). The depletion of importin β, however, did not affect the accumulation of the protein (Figure S4A,B). Furthermore, a version of the protein with a very large tag (EGFP_2_-GST-LRRC59 aa1-244) was confined to the cytoplasm (Figure S4C), demonstrating a lack of active nuclear import. Together, these results suggest that soluble versions of LRRC59 are not actively imported into the nucleus by an importin β-dependent mechanism (or in fact by any mechanism involving importin β-like transport receptors). Instead, they seem to passively diffuse into the nucleus, at least if they are small enough, where they are sequestered upon binding to nuclear structures. Indeed, the entire LRRC59-sequence up to the TMD is rather basic with an isoelectric point of 8.8, suggesting unspecific binding to chromatin.

### 2.6. The Size of the Cytoplasmic Domain Controls INM Localization of LRRC59

**Figure 6:**
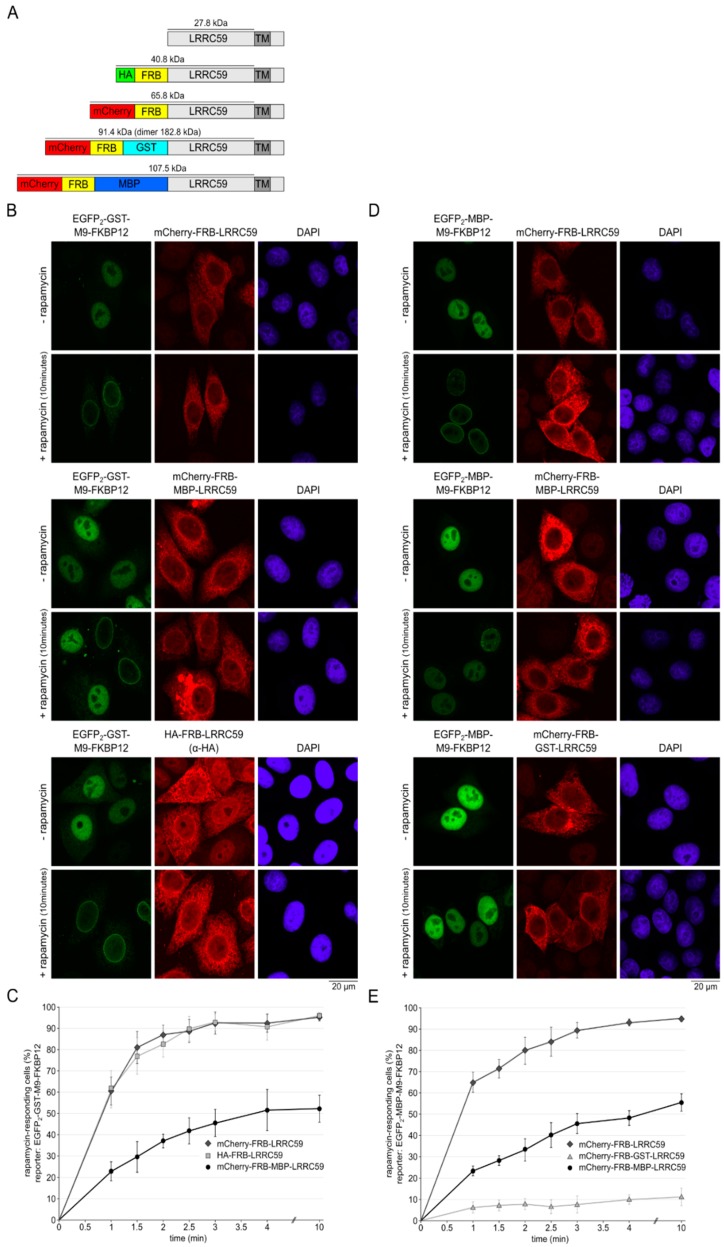
The size of the cytoplasmic domain of LRRC59 affects its targeting to the INM. (**A**) Schematic overview of LRRC59-constructs of different sizes. (**B**) HeLa cells were co-transfected with plasmids coding for EGFP_2_-GST-M9-FKBP12 and mCherry-FRB-LRRC59, mCherry-FRB-MBP-LRRC59 or HA-FRB-LRRC59, respectively. Plasmid concentrations were adjusted for similar expression levels of the different LRRC59 forms. After 48 h, cells were treated with or without rapamycin for 1–10 min, fixed and analyzed by fluorescence microscopy. Representative images for the 10 min-time point are shown. (**C**) Quantification of the experiment in (**B**). The percentage of cells responding to rapamycin with a recruitment of the reporter to the nuclear periphery was plotted against the time after addition of the drug. Error bars indicate the standard deviation from the mean of at least 4 independent experiments, counting 100 cells per time point. (**D**) HeLa cells were transfected with plasmids coding for EGFP_2_-MBP-M9-FKBP12 and mCherry-FRB-LRRC59, mCherry-FRB-MBP-LRRC59 and mCherry-FRB-GST-LRRC59, respectively. Plasmid concentrations were adjusted for similar expression levels of the different LRRC59 constructs. After 48 h, cells were treated as described in panel (**B**). (**E**) Quantification as in (**C**) of the experiment in (**D**). Error bars indicate the standard deviation from the mean of 5 independent experiments, counting 100 cells per time point.

Our results described so far did not point to an active, transport receptor-dependent targeting of LRRC59 to the INM. We therefore tested the alternative targeting model, where the protein would reach its destination by passive diffusion. One prediction of this model is the size-dependency of INM-targeting, resulting from small, perhaps peripheral channels of the NPC that could restrict passage of large membrane proteins. We therefore constructed versions of LRRC59 with cytoplasmic domains of different sizes and tested their INM-targeting in the rapamycin assay (Figure 6A). As shown in Figure 6B, three versions of LRRC59 (HA-FRB-LRRC59, mCherry-FRB-LRRC59 and mCherry-FRB-MBP-LRRC59) showed very similar subcellular localizations, with prominent ER-signals and also a rim around the nucleus, which could reflect targeting to the INM, the ONM or both. A clear difference, however, became apparent in the rapamycin assay: whereas cells expressing HA-FRB-LRRC59 or mCherry-FRB-LRRC59 reacted to rapamycin with a fast recruitment of the reporter protein EGFP_2_-GST-M9-FKBP12 to the INM, cells expressing mCherry-FRB-MBP-LRRC59 with its large cytoplasmic domain (107.5 kDa) responded much more slowly to the drug (Figure 6B,C). Finally, we tested an LRRC59-construct containing a GST-tag, which is expected to lead to the formation of large dimers [51]. To use this fusion protein in the rapamycin assay, we had to switch to a reporter protein lacking a GST-tag and used EGFP_2_-MBP-M9-FKBP12 instead of EGFP_2_-GST-M9-FKBP12. The localization of mCherry-FRB-GST-LRRC59 was very similar to that observed for mCherry-FRB-LRRC59 or mCherry-FRB-MBP-LRRC59 (Figure 6D). Strikingly, however, co-expressed EGFP_2_-MBP-M9-FKBP12 was hardly recruited to the INM upon addition of rapamycin to the cells, even after a prolonged incubation for 10 min (Figure 6D,E). For mCherry-FRB-LRRC59 and mCherry-FRB-MBP-LRRC59, very similar kinetics (Figure 6E) as described above (Figure 6C) were observed. This result suggested that only a small proportion of mCherry-FRB-GST-LRRC59 actually resided at the INM. It speaks against an active mechanism of protein transport to the INM, which should not be as strongly affected by protein size. Instead, our results argue for a diffusion-controlled translocation of LRRC59, from the ER via the ONM to the INM.

## 3. Discussion

### 3.1. Post-Translational Targeting of LRRC59 to ER-Membranes

In this study, we analyzed the molecular mechanisms that lead to insertion of LRRC59 into the ER-membrane and to its transport to the INM. The suggested topology of the protein with a single TMD and C-terminal luminal domain has previously been confirmed [31]. Compared to established tail-anchored proteins like Sec61β (with two amino acid residues behind the TMD) or emerin (11 amino acids), the 40-residues region behind the TMD of LRRC59 is rather long, suggesting that the protein could also be co-translationally inserted into the cellular membrane system, using the classic, SRP-dependent pathway. Our results now clearly show that LRRC59 can be inserted post-translationally, even with an artificial extension of 13 amino acids at its C-terminal end. We cannot, however, exclude the possibility that the endogenous protein also uses co-translational mechanisms. Which pathways could be used for post-translational insertion? Dominant negative WRB- or CAML-fragments (Figure 3A,B) or TRC40-depletion from reticulocyte lysates (Figure 3D,E) did not inhibit membrane insertion of LRRC59, in contrast to that of the model protein emerin. These observations suggest that the TRC-pathway is not essential for LRRC59 and are in line with the complex and diverse mechanisms that contribute to membrane integration in general. Under certain conditions, the SRP or Hsc70 proteins could also mediate post-translational events, circumventing the requirement for TRC40, WRB or CAML in the in vitro reaction [52]. Alternative mechanisms using other factors were described very recently [53,54,55,56]. Further studies will be needed to determine whether LRRC59 uses alternative, co- and/or post-translational pathways to reach the ER-membrane system in living cells.

### 3.2. Targeting of LRRC59 to the INM

Previous studies supporting active, NLS- and receptor-dependent transport of proteins to the INM were mainly performed in yeast [16,17,28]. In living mammalian cells, a systematic analysis of a large number of proteins suggested that Ran- and ATP-dependent processes could govern INM-localization of a subset of proteins [57], with the potential caveat that this requirement reflected active transport of INM-retention factors into the nucleus rather than active transport of the membrane protein itself. To circumvent these problems, very elegant in vitro assays were developed, where efficient transport of a membrane-bound reporter protein from the ER to the INM can only occur upon protease-mediated cleavage of a large, translocation-inhibiting protein fragment [19,20]. Together, these studies suggested that diffusion and retention is a major mechanism for INM-localization of membrane proteins. Accordingly, depletion of lamin A/C resulted in a loss of emerin from the INM and to its redistribution to the ER (data not shown). With respect to LRRC59, which was not affected by lamin A/C-depletion, the importin β-pathway was previously suggested to play a role in LRRC59-targeting [31]. Using several experimental approaches, however, we could not detect a strict requirement of the importin β-system in INM-targeting of LRRC59. Importantly, a soluble control cargo, whose nuclear import depends on importin β, was clearly affected by the siRNA-treatment and the dominant negative competitor protein Bimax2, demonstrating that importin β became rate-limiting under those conditions. At present, the reasons for the different results presented before [31] and those described here remain unclear. Different knock-down efficiencies appear rather unlikely, since we could not observe effects on LRRC59 in cells with the highest possible importin β knockdown that was still compatible with cell growth and survival. In an independent experimental approach, nuclear accumulation of a soluble version of LRRC59 lacking the TMD occurred by diffusion and retention, independent of importin β (Figure S4). Again, this speaks against the involvement of active, factor-mediated transport.

Previous studies have suggested a size-limit of ~60–75 kDa for efficient translocation of membrane proteins from the ONM to the INM [20,21,24,57]. For LRRC59, we also observed a clear size-limit, albeit somewhat shifted to higher molecular weights (Figure 6). Our LRRC59-constructs with cytoplasmic/nuclear regions of 40.8 kDa (HA-FRB-LRRC59) and 65.8 kDa (mCherry-FRB-LRRC59) allowed very similar recruitment of the nuclear reporter to the nuclear envelope. mCherry-FRB-MBP-LRRC59 with a cytoplasmic/nuclear region of 107.5 kDa could still reach the INM, but clearly less efficiently. mCherry-FRB-GST-LRRC59 with an expected dimer-size of 182.8 kDa for the cytoplasmic region, was excluded from the INM. These results agree with a diffusion-governed mechanism that does not involve active transport mechanisms. The exact size-limit for translocation may vary from one specific protein to the other, depending on its shape and the artificial tags used in the experiment. 

The major function of LRRC59 is unknown and at present, it is unclear if LRRC59 in fact has a role in the nucleus. Interestingly, it was suggested that LRRC59 is involved in nuclear import of fibroblast growth factor 1 [31] and the cancerous inhibitor of PP2A (CIP2A) [32]. If such a transport-related function also involves a retention mechanism that would sequester a certain proportion of LRRC59 at the INM remains to be investigated.

## 4. Materials and Methods 

### 4.1. Plasmids and Constructs

Plasmids coding for pmCherry-FRB, pGEM3Z-emerin-opsin, pET328-HZZ-emerin-opsin, [40] and pET328-HZZ-cytochrome b5-opsin [38,44] were described previously. 

The human LRRC59-sequence was amplified by PCR using 5′-tttCTCGAGctaccatgaccaaggccggtagc-3′ and 5′-tttGAATTCtcactgctgagagtcggtc-3′ or 5′-tttCTCGAGatgaccaaggccggtagc-3′ and 5′-tttGAATTCtcactgctgagagtcggtc-3′ as primers and pcDNA3-LRRC59 (obtained from Dr. Antoni Wiedlocha, Oslo University Hospital) as template, and cloned via XhoI and EcoRI into pmCherry-C1 or a modified pmCherry-C1-vector containing an FRB-cassette inserted via BglII and XhoI. MBP- and GST-sequences were amplified from appropriate vectors using 5′-aaaACTCGAgatgaaaatcgaagaaggtaaactg-3′ and 5′-aaaACTCGAgattgttattgttgttgttgttcgag-3′ or 5′-aaaCTCGAGtcccctatactaggttattgg-3′ and 5′-aaaCTCGAGttttggaggatggtcgcc as primers and inserted into pmCherry-FRB-LRRC59 using XhoI to generate pmCherry-FRB-MBP-LRRC59 and pmCherry-FRB-GST-LRRC59, respectively. For HA-FRB-LRRC59, the LRRC59-sequence was PCR amplified using 5′-aaaaCTCGAGaaatgaccaaggccggtagc-3′ and 5′-aaaaTCTAGAtcactgctgagagtcggtc-3′ as primer and pcDNA3-LRRC59 as template and cloned via XhoI and XbaI into a modified pEGFP-C1 vector, where the EGFP-sequence had been replaced by a sequence coding for HA-FRB.

To create pEGFP_2_-GST-M9-FKBP12, the M9-sequence was amplified by PCR using 5′-tttGGTACCgggaattacaacaatcagtct-3′ and 5′-tttGGATCCatagccaccttggtttcgtg-3′ as primers and cloned into pEGFP_2_-GST-cNLS-FKBP12 [40] via BamHI and KpnI, replacing the cNLS-sequence. To create pEGFP_2_-MBP-M9-FKBP12, three PCR-fragments (XhoI-MBP-HindIII using 5′-aaaaaCTCGAGaaatgaaaatcgaagaaggtaaactg-3′ and 5′-aaaAAGCTTattgttattgttgttgttgttcgag-3′ as primers; HindIII-M9-EcoRI using 5′-aaaAAGCTTatggggaattacaacaatcagtcttc-3′ and aaaaaGAATTCatagccaccttggtttcgtg-3′; EcoRI-FKBP12-BamHI using 5′-aaaaaGAATTCatggctagcggagtgcagg-3′ and 5′-aaaGGATCCtcattccagttttagaagctccacatc-3′) were cloned simultaneously into pEGFP_2_-C1 via XhoI and BamHI, which contains a second EGFP-sequence inserted into the NheI-site of pEGFP-C1. To generate pcDNA3.1-WRB-FRB-HA, PCR-fragments containing the WRB-sequence were obtained using pRK-HA-WRB as template (a kind gift from Dr. Fabio Vilardi, Göttingen) and 5′-ctaGCTAGCatgagctcagccgcggccgac-3′ and 5′-cccAAGCTTgctgaacggatgaagcacaat-3′ as primers and the FRB-sequence as template and 5′-cccAAGCTTgagatgtggcatgaaggcc-3′ and 5′-gcGGATCCctgctttgagattcgtcggaa-3′ as primers. PCR products were inserted via NheI and BamHI into pcDNA3.1(+) containing a C-terminal HA-tag inserted via EcoRI and XhoI. To generate pcDNA3-FLAG-Bimax2, the sequence of Bimax2 [50] (a kind gift from Dr. Dorothee Dormann, Munich) was PCR amplified using the FLAG-sequence-containing primers 5′-aaaGGATCCatggactacaaagacgatgacgacaagatgcggaggagacgacggagaa-3′ and 5′-aaaaaGAATTCtcagtccagccttctccgcttc-3′ and cloned into pcDNA3 (Invitrogen) via BamHI and EcoRI. To create pcDNA3-NES-mTagBFP2-cNLS, the BFP-sequence was amplified using yeast mTagBFP2 (a kind gift from Dr. Blanche Schwappach, Göttingen) as template, the NES-sequence-containing primer 5′-aaaaaGAATTCatgctaccaccactagaacgactgacactaatggtgtctaagggcgaaga-3′ and the cNLS-sequence containing primer 5′-aaaaCTCGAGtcaaactttcctctttttctttggattaagcttgtgccccagttt-3′, and cloned into pcDNA3 via EcoRI and XhoI. The sequence of LRRC59 aa1-244 was amplified by PCR using 5′-aaaaCTCGAGaaatgaccaaggccggtagc-3′ and 5′-aaaaaGAATTCtcaggaacgagtgtgcttccg-3′ as primers and cloned into pEGFP-C1 (Clontech) via XhoI and EcoRI. To create pEGFP_2_-GST-LRRC59 aa1-244, the LRRC59-fragment was amplified by PCR using 5′-aaaaaGAATTCaatgaccaaggccggtagc-3′ and 5′-aaaaTCTAGAttaggaacgagtgtgcttccg-3′ as primers and cloned via EcoRI and XbaI into a modified pEGFP_2_-vector with the GST-sequence inserted via BglII and XhoI into pEGFP_2_. For pET328-HZZ-LRRC59-opsin, the LRRC59 sequence was amplified by PCR using 5′-tttGGTACCaccatgaccaaggccggtagc-3′ and 5′-tttAAGCTTtatcagcccgtcttgttggagaaaggcacgtagaagtttgggccctgctgagagtcggtctg-3′ containing the sequence of the opsin-tag [58], and cloned into a modified pET-vector (pET328) using KpnI and HindIII. Primers were ordered from Sigma-Aldrich, Munich, Germany. All constructs were verified by sequencing.

### 4.2. Cell Culture, Plasmid- and siRNA- Transfection and Immunofluorescence Microscopy

HeLa p4 cells [59] and U2OS cells were grown in DMEM (Thermo Fisher Scientific, Waltham, USA) supplemented with 10% (*v*/*v*) FBS Superior (Biochrom, Berlin, Germany), 100 U/mL penicillin, 100 µg/mL streptomycin and 2 mM L-glutamine (Thermo Fisher Scientific) under 5% CO_2_ at 37 °C. Cells were tested for mycoplasma on a regular basis. For microscopy-based cell assays, cells were grown on non-coated coverslips and transiently transfected with plasmid DNA and/or siRNA using the calcium phosphate method [60]. For knock-down of importin β, cells were transfected with siRNAs against importin β (siRNA1, 5′-ACAGUGCCAAGGAUUGUUA[dT][dT]-3′; Eurofins (Hamburg, Germany) and siRNA2, 5′-CUGGAAUCGUCCAGGGAUUAA[dT][dT]-3′; Sigma; 50 nM each) or 100 nM non-targeting control siRNA (Dharmacon, Lafayette, USA, D-001810-01-50). For knock-down of LRRC59, 100 nM siRNA 5′-GGAGUAUGAUGCCCUCAAAG[dT][dT]-3′ (Sigma-Aldrich) was used.

Cells were fixed using 3.7% (*v*/*v*) formaldehyde in PBS and either analyzed directly by fluorescence microscopy or after indirect immunostaining. For this, cells were permeabilized with 0.5% TritonX-100 in PBS at room temperature for 5 min, followed by blocking with 3% BSA in PBS for 20 min. Primary antibodies were diluted in 3% BSA in PBS and added to the sample at room temperature for 1.5–2.5 h. After washing with PBS, cells were incubated with secondary antibodies diluted in 3% BSA in PBS at room temperature for 1–1.5 h. For detection of endogenous LRRC59, 3 µg/mL of anti-LRRC59 antibody was diluted in blocking buffer supplemented with 0.05% Triton-X-100.

The following antibodies were used: mouse anti-myc (Santa Cruz, Dallas, USA, sc-40; 1:200), rabbit anti-emerin (Proteintech, Manchester, UK, 10351-1-AP; 1:500), rabbit anti-LRRC59 (Sigma-Aldrich, HPA030829; 3 µg/mL, 1:100), mouse anti-HA (Covance, Princeton, USA, MMS-101P; 1:1000), mouse anti-lamin A/C (Abcam, ab40567; 1:250), rabbit anti-importin β ([61]; 1:500), mouse anti-FLAG (Sigma, F3165; 1:3000), donkey anti-mouse Alexa Fluor 594, donkey anti-rabbit Alexa Fluor 594, donkey anti-rabbit Alexa Fluor 488, donkey anti-mouse Alexa Fluor 488, goat anti-mouse Alexa Fluor 647 and goat anti-rabbit Alexa Fluor 647 (Molecular Probes, Eugene, OR, USA).

Cells were mounted with Mowiol 4-88 (13.3% (*w*/*v*) (Calbiochem, Merck Milipore, Burlington, USA), 33.3% (*w*/*v*) glycerol, 133 mM Tris-HCl, pH 8.5) containing 1 µg/mL DAPI, except for cells transfected with pcDNA3-NES-mTagBFP2-cNLS. 

For quantitative analysis, an Axiovert 200M fluorescence microscope (Zeiss, Oberkochen, Germany) with a 63x Plan-Neofluar 1.3 NA water-corrected objective and appropriate filter settings was used. Images were taken using the LSM 510-META confocal laser scanning mode and processed using ImageJ.

### 4.3. SDS-PAGE, Ponceau S Staining and Western Blotting

Proteins were analyzed by SDS-PAGE, followed by Western blotting and, for loading controls, Ponceaus S staining using standard methods.

For protein detection, mouse anti-opsin ([58] 1:1000), rabbit anti-emerin (Proteintech, 10351-1-AP; 1:1000), rabbit anti-LRRC59 (Sigma, HPA030829, 1:250), rabbit anti-calnexin (Enzo Life Sciences, Lörrach, Germany, ADI-SPA-860; 1:1000), rabbit anti-PDI (Cell Signaling, Cambridge, USA, 3501; 1:1000), mouse anti-TRC40 (Sigma, WH0000439M3-100UG; 1:1000), guinea pig anti-CAML (Synaptic Systems, Göttingen, Germany, 359004; 1:1000), rabbit anti-importin β ([61]; 1:1000), and rabbit anti-alpha-tubulin (Proteintech, 11224-1-AP; 1:1000) were used. For detection of secondary antibodies (LI-COR IRDye 800CW goat anti-mouse; IRDye 800CW donkey anti-mouse; IRDye 800CW donkey anti-rabbit; IRDye 680CW donkey anti-rabbit; IRDye 680CW donkey anti-mouse, IRDye 680CW donkey anti-guinea pig), the Odyssey system (LI-COR, Bad Homburg, Germany) was used. For comparison, the protein of interest was normalized to the signal of alpha-tubulin using Image Studio Lite 2. 

### 4.4. Microsome Integration Assay

The procedure of coupled in vitro transcription and translation in a microsome-integration assay was described previously [40,41,45]. Briefly, pGEM3Z-emerin-opsin, pET328-HZZ-tev-emerin-opsin or pET328-HZZ-tev-LRRC59-opsin were expressed in vitro using 8.8 µL rabbit reticulocyte lysate (TnT T7 Quick Coupled Transcription/Translation System, Promega, Madison, WI, USA), supplemented with methionine and 200 ng of plasmid-DNA. Reactions were incubated at 30 °C for 90 min. Where indicated, puromycin (Sigma-Aldrich, St. Lois, MO, USA) was added to a final concentration of 2.5 mM and reactions were incubated for 10 min prior to the addition of purified dog pancreas rough microsomes [62], trypsin- (T-RK) [45] or EDTA/high-salt-treated (EK-RM) microsomes for additional 60 min. Reactions were stopped by the addition of 50 µL of SDS-loading buffer (4% SDS, 125 mM Tris pH 6.8, 10% glycerol, 0.02% bromophenol blue, 10% β-mercaptoethanol). 10–25% of the reaction was analyzed by SDS-PAGE and immunoblotting.

To confirm glycosylation of opsin-tagged LRRC59, 25% of an insertion reaction was mixed with glycoprotein denaturing buffer (5% SDS, 0.4 M DTT) and denatured at 100 °C for 10 min. Either 1 µL of peptide-N-Glycosidase F (PNGase F, 500 units/µL; New England BioLabs, Ipswich, MA, USA) or water was added to the reaction supplemented with 10% reaction buffer (0.5 M sodium phosphate, pH 7.5) and 1% NP-40 at 37 °C for 60 min. 

Where indicated, MBP-WRBcc [41] or GST-CAML-N [42] were added to a final concentration of 5 or 10 µM, 10 min before the addition of RM. Purified MBP-WRBcc and GST-CAML-N were a kind gift from Dr. Jhon Rivera Monroy (Göttingen).

The depletion of TRC40 from rabbit reticulocyte lysate was described previously [63]. Briefly, 20 µL of Protein A Sepharose 4 Fast Flow (GE Healthcare, Chicago, IL, USA), equilibrated in cold PBS, were incubated with 15 µL of rabbit anti-TRC40 [45] or 1.5 µg of rabbit IgG (Sigma-Aldrich) at 4 °C for 60 min. After washing steps using cold PBS, 120 µL of reticulocyte lysate were added to the immobilized antibodies at 4 °C for 60 min. The in vitro transcription and translation microsome integration assay using the TRC40- or mock-depleted lysate was performed as described above. For quantification, line scans were performed using ImageJ and membrane insertion efficiencies were expressed as the percentage of the glycosylated protein compared to the sum of the glycosylated and the non-glycosylated form. Values were normalized to control reactions. 

### 4.5. Rapamycin Induced Dimerization Assay

To check for INM-localization of a transmembrane protein, HeLa p4 cells grown on non-coated coverslips were transiently transfected with the reporter pEGFP_2_-GST-M9-FKBP12 or pEGFP_2_-MBP-M9-FKBP12 and an FRB-containing protein of interest and, in some experiments, with siRNAs and pcDNA3-FLAG-Bimax2 and pcDNA3-NES-mTagBFP2-cNLS. After 48 h of knock-down and/or overexpression, coverslips were transferred into a humidity chamber at room temperature. Cells were incubated with 200 nM rapamycin (Sigma-Aldrich) diluted in PBS for 1-10 min and immediately fixed using 3.7% formaldehyde in PBS. If necessary, immunofluorescence staining was performed prior fluorescence microscopy. For the analysis of rapamycin induced reporter response, at least 100 cells were counted per condition and categorized as “responding” or “not responding”. At least four independent experiments were performed per condition.

## 5. Conclusions

INM-targeting of LRRC59 had been suggested to require the classic transport machinery that is also involved in nuclear import of soluble proteins. We conclude, however, that LRRC59, which can be inserted into membranes by post-translational mechanisms, reaches the INM mainly by passive diffusion.

## Supplementary Materials

Supplementary materials can be found at http://www.mdpi.com/1422-0067/20/2/334/s1.

## Abbreviations

CAML	Ca^2+^-modulating cyclophilin ligand
CIP2A	cancerous inhibitor of PP2A
cNLS	classical nuclear localization signal
ER	endoplasmic reticulum
EK-RM	EDAT and high salt treated rough microsomes
FKBP12	12 kDa FK506 binding protein
FG repeats	phenylalanine-glycine repeats
FRB	FKBP rapamycin binding domain
INM	inner nuclear membrane
LBR	Lamin B receptor
LRRC59	leucine-rich repeat-containing protein 59
NES	nuclear export sequence
NLS	nuclear localization signal
NPC	nuclear pore complex
ONM	outer nuclear membrane
PNGase F	Peptide-N-glycosidase F
RM	rough microsomes
T-RM	trypsin-treated rough microsomes
TA	tail-anchored
TMD	Transmembrane domain
TRC40	ATPase ASNA1
WRB	tryptophan-rich basic protein
