# Supplementary material for: Targeting of LRRC59 to the Endoplasmic Reticulum and the Inner Nuclear Membrane
Source: Int J Mol Sci. 2019 Jan 15;20(2):334. doi: 10.3390/ijms20020334 (PMC6359192; doi:10.3390/ijms20020334)
Supplement: Supplementary file 1 [file ijms-20-00334-s001.pdf]

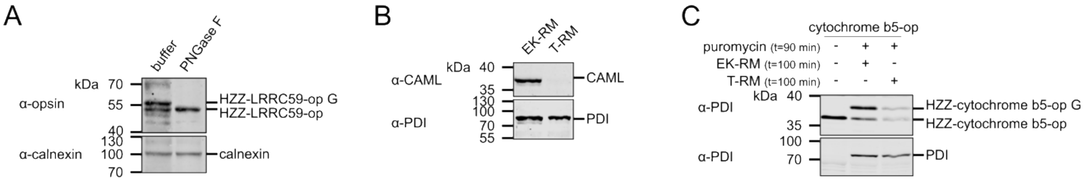

Figure S1. Posttranslational membrane insertion of LRRC59 (related to figures 2 and 3). (A) N-linked glycosylation of HZZ-LRRC59-op. HZZ-LRRC59-op was produced and modified as in figure 2B and subjected to treatment with PNGase F or buffer as indicated. Proteins were analyzed by SDS-PAGE and Western-blotting, using an antibody against the opsin-tag. Calnexin was used as a loading control. (B) Comparison of trypsin- (T-RM) and EDTA/high salt-treated (EK-RM) microsomes. Microsomes were mixed with SDS-loading buffer and analyzed by SDS-PAGE followed by Western-blotting, detecting CAML and, as loading control, PDI. (C) HZZ-cytochrome b5-opsin was produced in a coupled *in vitro* transcription/translation reaction and incubated with EDTA/high salt- (EK-RM) or trypsin- (T-RM) treated microsomes, with (+) or without (-) the addition of puromycin, as indicated. Proteins were analyzed by SDS-PAGE followed Western-blotting using antibodies against the opsin-tag and PDI.

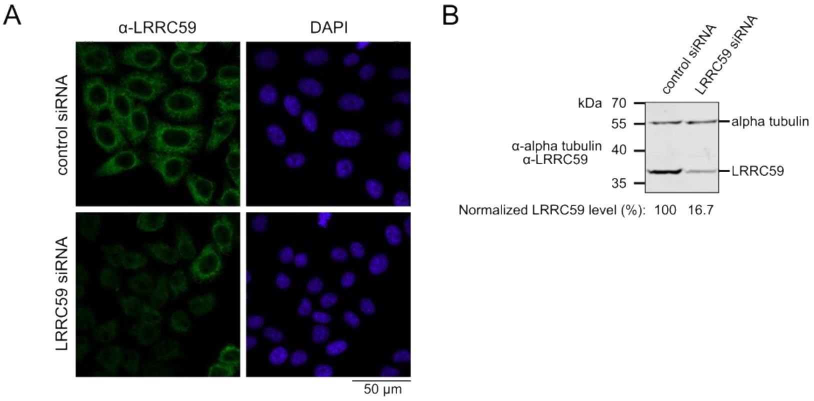

Figure S2. Specificity of the anti-LRRC59 antibody. HeLa cells were transfected with control siRNAs or siRNAs against LRRC59, fixed after 48 h and analyzed by indirect immunofluorescence using an antibody against LRRC59 (A) or analyzed by SDS-PAGE and immunoblotting using antibodies against LRRC59 and alpha-tubulin (B). Relative LRRC59-levels were normalized to alpha-tubulin.

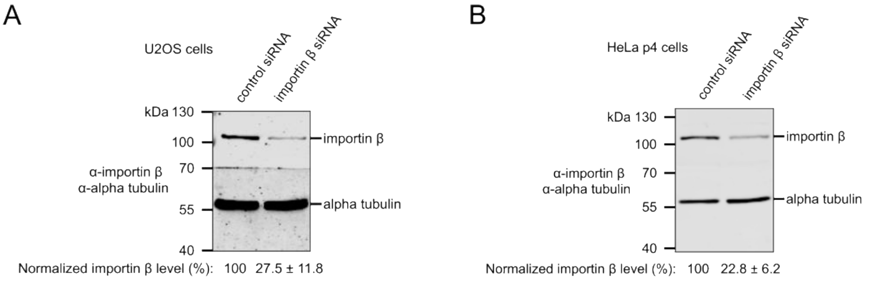

Figure S3. Knock-down of importin β (related to Figure 5). U2OS cells (A) or HeLa cells (B) were treated with control siRNAs or siRNAs against importin β and analyzed by SDS-PAGE followed by Western-blotting, detecting importin β and alpha-tubulin. The standard deviations from the mean of importin β levels in four (A) or nine (B) experiments, normalized to alpha-tubulin, are indicated.

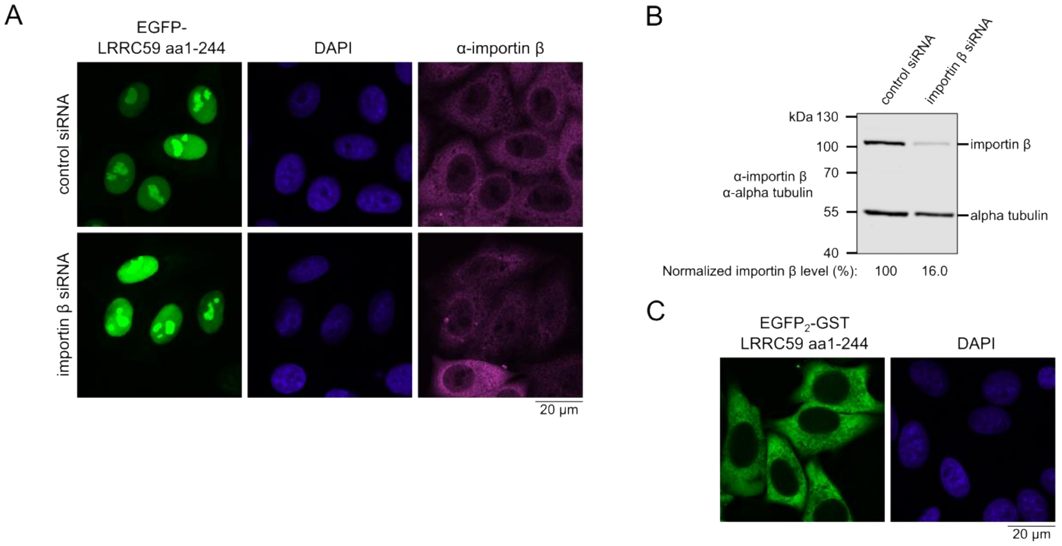

Figure S4. Size-dependent localization of LRRC59 lacking a TMD. (A) HeLa cells were transfected with a plasmid coding for EGFP-LRRC59 aa1-244 and control siRNAs or siRNAs against importin β. After 48 h, cells were fixed and analyzed by indirect immunofluorescence using antibodies against importin β. (B) Cells were treated as in (A) and analyzed by SDS-PAGE and Western-blotting, detecting importin β and alpha-tubulin. The importin β level was normalized to that of alpha-tubulin. (C) HeLa cells were transfected with a plasmid coding for $EGFP_2$-GST-LRRC59 aa1-244. After 48 h, cells were fixed and analyzed by fluorescence microscopy.
